# Correlation Analysis of Clinical, Imaging, and Genetic Etiologies in Pediatric Hereditary Cerebellar Atrophy: A Single‐Center Study

**DOI:** 10.1002/mgg3.70258

**Published:** 2026-07-06

**Authors:** Luyao Jin, Shuling Chen, Ying Liu, Renyi She, Wei Jiang

**Affiliations:** ^1^ Department of Rehabilitation Children's Hospital of Chongqing Medical University, National Clinical Research Center for Children and Adolescents' Health and Diseases, Ministry of Education Key Laboratory of Child Development and Disorders Chongqing City China; ^2^ Chongqing Key Laboratory of Child Neurodevelopment and Cognitive Disorders Chongqing City China; ^3^ People's Hospital of Kaizhou District Chongqing City China; ^4^ Chongqing Maternal and Child Health Hospital Chongqing City China; ^5^ Department of Rehabilitation Yibin Hospital Affiliated to Children's Hospital of Chongqing Medical University Yibin Sichuan China

**Keywords:** ataxia, cerebellar atrophy, children, gene mutation, hereditary

## Abstract

**Objective:**

To investigate the associations among clinical features, neuroimaging findings, and genetic data in children with hereditary cerebellar atrophy (CA).

**Method:**

A cohort of 102 pediatric patients diagnosed with hereditary CA was enrolled at the Children's Hospital of Chongqing Medical University (2015–2024). Univariate and multivariate analyses assessed clinical‐neuroimaging‐genetic correlations.

**Results:**

Earlier onset correlated with prematurity (*p* = 0.039) and negative family history (*p* = 0.042); diagnostic delay with unremarkable perinatal history (*p* = 0.038). Motor delay was more prevalent in males (100% vs. 91.1%). Gross motor scores were lower in membrane transporters (26.78 ± 17.90, *p* = 0.027) and metabolic diseases (28.09 ± 26.26, *p* = 0.025) compared to cytoskeletal proteinopathies (47.81 ± 13.55). Multivariate analysis identified age at onset and diagnostic delay as independent predictors of motor and cognitive development delay and enzymopathies/glycoprotein disorders for global developmental delay (OR = 4.4, *p* = 0.042). Early onset (≤ 6 months) elevated the risk of ataxia (OR = 6.75, *p* = 0.021). Atrophy severity independently predicted motor and cognitive impairment.

**Conclusion:**

Preterm birth, male sex, and negative family history predicted earlier onset, urging early neuroimaging. Early onset and severe/complex cerebellar atrophy indicated poorer prognosis. While ataxia was uncommon overall, onset ≤ 6 months increased its risk. Metabolic disorders contributed to significant motor deficits, underscoring the need for early genetic testing and targeted management.

## Graphical abstract

We identified predictors of early onset and poor prognosis in childhood cerebellar atrophy. Our findings advocate for a shift towards early, targeted diagnostic strategies based on genetic and imaging profiles.

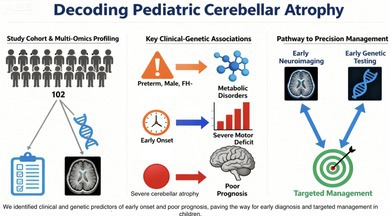

## 1 Introduction

Cerebellar atrophy (CA) is a rare neuroimaging feature in pediatric neurology, characterized by widening of the cerebellar cortical sulci due to tissue loss, with initially normal cerebellar structure and posterior fossa size (Poretti et al. 2008). Diagnosis relies on magnetic resonance imaging (MRI). The cerebellum is involved not only in regulating gait, posture, balance, and coordination of goal‐directed movements (Ataullah et al. 2024; Ghez and Thach 2000), but also extensively participates in cognitive, behavioral, and emotional processing (Marek et al. 2018; Chao et al. 2023; Picerni et al. 2022). Pediatric CA has diverse etiologies, including genetic, acquired, and idiopathic. Hereditary CA encompasses both familial inheritance and de novo mutations, demonstrating high phenotypic and genetic heterogeneity, and involves multiple genes and molecular mechanisms (Martínez‐Rubio et al. 2023; Martínez‐Rubio et al. 2022; Coarelli et al. 2023). Advances in genetic technologies since the 1990s have identified numerous related genes. Nonetheless, variability in mutation type, age of onset, clinical manifestations, treatment, and prognosis continues to pose diagnostic and genetic testing challenges.

Recent studies on hereditary CA in children are largely confined to data from 2015. We aimed to explore associations among clinical, imaging, and genetic data in a Chinese pediatric CA dataset using correlation and multivariate regression analyses, with the goal of informing diagnosis and long‐term management.

## 2 Subjects and Methods

### 2.1 Study Population

A retrospective analysis was conducted on the clinical, auxiliary examination, and genetic data of 102 pediatric patients with cerebellar atrophy, diagnosed by genetic testing at the Children's Hospital affiliated with Chongqing Medical University between January 2015 and January 2024.

Indications for brain MRI and genetic testing: Brain MRI was performed in children with early‐onset motor delay, ataxia, dystonia, gait abnormalities, or developmental delay; upon confirmation of cerebellar atrophy and exclusion of acquired causes, genetic testing was pursued for suspected hereditary etiologies.

### 2.2 Methodology

#### 2.2.1 Inclusion and Exclusion Criteria

*Inclusion criteria*: (1) Following the internationally unified diagnostic criteria, imaging findings indicating reduced cerebellar volume, cortical thinning, and widened folia and fissures. Enlargement of the pericerebellar CSF space and fourth ventricle is supportive but not required; (2) Genetically confirmed hereditary etiology; (3) Age between 0 and 16 years; (4) Pediatric neurologists assessed the imaging‐clinical correlation. Patients were included if their clinical features matched the distribution/severity of cerebellar atrophy on MRI, or if genetic testing was clearly positive (pathogenic/likely pathogenic) with imaging‐confirmed atrophy even without overt cerebellar symptoms. Asymptomatic cases with VUS were excluded.

*Exclusion criteria*: Acquired cerebellar atrophy (e.g., cerebrovascular events, traumatic brain injury and space‐occupying lesions).

#### 2.2.2 Data Collection

Clinical data were collected from 102 pediatric patients genetically diagnosed with hereditary CA: (1) Medical history: demographic information (age, sex), disease course (age at onset, age at diagnosis), family history, and clinical manifestations; (2) Physical examination: muscle strength, muscle tone, and pathological reflexes (pyramidal and extrapyramidal signs); (3) Auxiliary examinations: cranial MRI, neuropsychological assessments, and genetic testing.

This study was approved by the Ethics Committee of the Children's Hospital affiliated with Chongqing Medical University (Ethics Approval No. 349 for the year 2023), and written informed consent has been obtained from the guardians of the participants.

#### 2.2.3 Genetic Testing Methods and Interpretation of Results

This study utilized trio‐based whole‐exome sequencing, copy number variation analysis, capillary electrophoresis‐based fragment analysis, and clone sequencing to identify gene variants associated with hereditary cerebellar atrophy. Procedures included sample preparation, library construction, sequencing, data processing, variant calling, and pathogenicity evaluation. Variants were classified according to the 2015 American College of Medical Genetics and Genomics (ACMG) standards and guidelines, combined with data from public databases including ClinVar, HGMD, and gnomAD. Variants classified as pathogenic (P), likely pathogenic (LP), or of uncertain significance were confirmed by Sanger sequencing. Capillary electrophoresis and clone sequencing were used to detect polynucleotide repeat expansions. For variants classified as P/LP according to ACMG guidelines, if an autosomal recessive condition presented with a P/LP variant in one allele and a VUS in the other, we coordinated with the genetic testing laboratory to further evaluate the VUS by integrating multiple lines of evidence, including MEF values, evolutionary conservation, and functional domain localization, to determine its clinical relevance. Genetic diagnoses were established based on three criteria: (1) Phenotypic consistency, (2) Co‐segregation evidence, and (3) Biological pathogenicity.

#### 2.2.4 Statistical Methods

Data were analyzed using R software (4.4.1). Exploratory analyses of demographic, imaging, clinical, and genetic data were conducted to identify candidate variables for multivariate logistic regression modeling. Continuous variables conforming to normal distribution as assessed by Shapiro–Wilk test were presented as mean ± standard deviation (x̄ ± SD) and compared using ANOVA with LSD *t*‐test for post hoc pairwise comparisons, while non‐normally distributed data were expressed as median (interquartile range) [*M* (P25, P75)] and analyzed using Kruskal–Wallis rank test or Wilcoxon signed‐rank test. Categorical variables were presented as percentages and compared using *χ* ^2^ test or Fisher's exact test. Statistical significance was set at *p* < 0.05. Variables with significant differences were included in a multiple linear regression model with a significance level of *α* = 0.05.

## 3 Results

### 3.1 Participant Demographics

The baseline characteristics of the 102 children with hereditary CA, including age at onset, prematurity, family history, and perinatal history are summarized in Table 1. Notably, family history differed significantly among subgroups, with the cohort predominantly comprising patients with a negative family history.

**TABLE 1:** Demographic characteristics of 102 cases.

	*n* (%) or median (IQR)	*p* (< 0.05)
*Sex*		
Male	57/102 (55.88%)	0.235
Female	45/102 (44.12%)	
*Gestational age*		
Preterm Birth	14/66 (21.21%)	0.152
Term	52/66 (78.79%)	
*Prenatal & birth history*		
Positive	43/102 (41.16%)	0.113
Negative	59/102 (57.84%)	
*Family history*		
Positive	26/102 (25.49%)	< 0.001
Negative	76/102 (74.51%)	
*Age*		
Age at onset	8 (3–15)	
Age at diagnosis	19 (6.75–38.50)	

### 3.2 Clinical Manifestations

Age at onset was categorized into five stages: (1) ≤ 6 months; (2) 6–12 months; (3) 12–36 months; (4) 36–72 months; and (5) > 72 months. Due to the limited sample size, stages 4 and 5 were combined for analysis. The final four categories were assigned ordinal scores of 1 through 4 for regression analysis.

All patients underwent cranial MRI, and the severity of cerebellar atrophy was classified as mild, moderate, or severe based on cerebellar volume reduction, cortical thinning, and enlargement of sulci and fissures (see Figure S1). Classification of topographic patterns was based on visual assessment of imaging appearance, not on quantitative volumetric measurements, and followed established frameworks (Poretti et al. 2015; Zhao et al. 2018): (1) CA ± dentate nucleus/cerebellar white matter involvement; (2) CA + supratentorial white matter lesions; (3) CA + cortical dysplasia; (4) CA + basal ganglia/thalamic abnormalities; (5) CA + brainstem atrophy; (6) Multisystem degeneration (≥ 2 extracerebellar involvements). Groups 4 and 5 were merged into a single category for analysis, as they represent contiguous neuroanatomical regions and had limited individual sample sizes.

**FIGURE 1:**
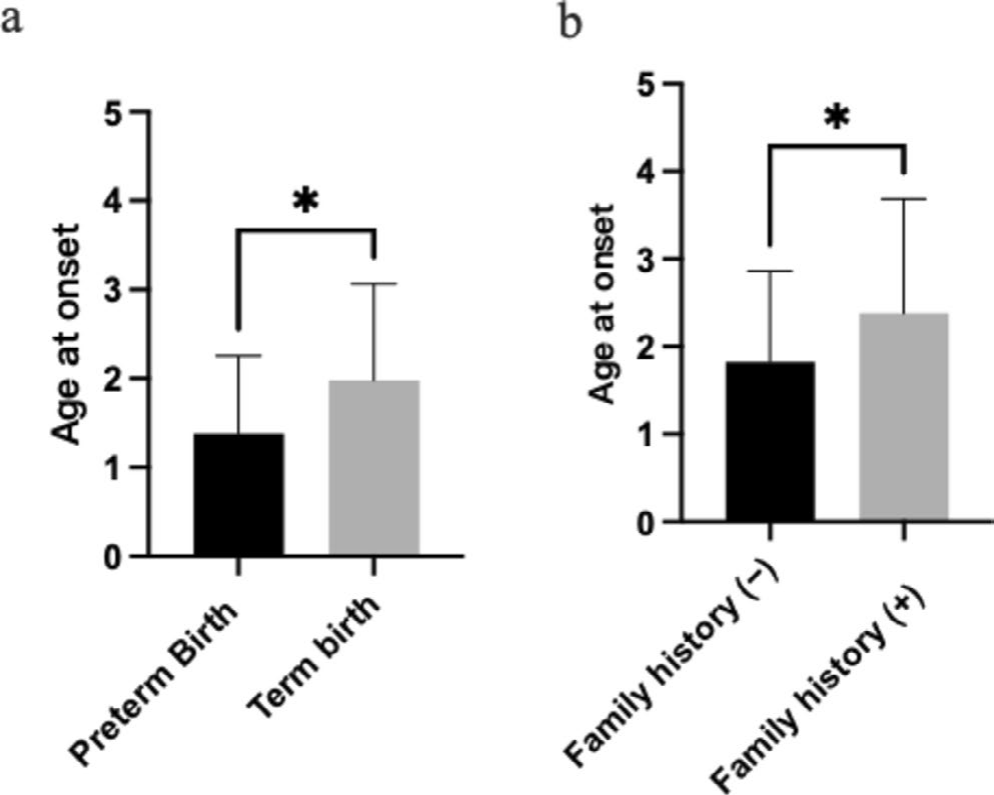
(a) Age at onset by gestational age; (b) Age at onset by family history.

Preterm birth or negative family history presented with a younger onset age (median: 1.00 [1.00–1.50] vs. 2.00 [1.00–3.00] months, *p* = 0.039; and 1.00 [1.00–2.75] vs. 2.00 [1.00–2.75] months, *p* = 0.042, respectively; Figure 1a,b). In contrast, a positive perinatal history was associated with a shorter diagnostic delay (2.00 [1.00–3.00] vs. 3.00 [2.00–4.00] months, *p* = 0.038).

Males showed higher motor delay rates than females (100% vs. 91.1%, *p* = 0.035). Low birth weight (< 2.5 kg) infants predominantly had hypertonia (83.3%), while normal weight infants showed normal tone (32.1%) or hypotonia (35.7%) (*p* = 0.021) (Table 2). A statistically significant difference in the incidence of motor developmental delay was observed among patients with different diagnostic delay durations (*p* = 0.023); all patients with disease duration ≤ 36 months exhibited motor developmental delay (Table 3).

**TABLE 2:** Univariate analysis of baseline characteristics and clinical manifestations.

	Age at onset (month)					Diagnostic delay (months)					Motor developmental delay			Global developmental delay			Ataxia			Muscle tonic disorder				
	< 6	6–12	13–36	> 36	*p*	< 6	6–12	13–36	> 36	*p*	+	−	*p*	+	−	*p*	+	−	*p*	Normal	Hypotonia	Hypertonia	Dystonia	*p*
*Sex*																								
Male	28	14	9	5	0.333	11	10	19	13	0.747	57	0	**0.035**	42	15	0.142	28	29	0.358	15	24	13	5	0.142
Female	19	10	8	7		13	2	14	14		41	4		27	18		18	27		9	16	19	1	
*Gestational age*																								
Preterm Birth	10	2	0	1	**0.039**	4	1	2	6	0.454	12	1	0.2	9	4	0.715	9	4	0.213	2	7	4	0	0.334
Term Birth	22	14	11	4		13	6	19	11		52	0		42	11		27	26		17	17	15	4	
*Birth weight (kg)*																								
< 2.5	4	2	0	0	0.208	3	0	2	1	0.462	6	0	0.741	3	3	0.141	4	2	0.438	0	1	5	0	**0.021**
≥ 2.5	25	14	12	5		13	8	17	16		55	1		45	12		28	28		18	20	14	5	
*Prenatal & birth history*																								
Positive	23	9	9	2	0.193	13	9	11	9	**0.038**	57	2	0.746	37	22	0.212	23	23	0.146	12	25	18	4	0.769
Negative	24	15	8	10		11	3	22	18		41	2		32	11		36	20		12	15	14	2	
*Family history*																								
Positive	8	8	4	6	**0.042**	5	2	11	6	0.721	72	4	0.233	52	24	0.775	35	11	0.74	17	28	29	2	**0.016**
Negative	39	16	13	6		19	10	22	21		26	0		17	9		41	15		7	12	3	4	

*Note:* +, present; −, absent; Data are presented as number. Bold coefficients indicate statistical significance at *p* < 0.05 (tow‐tailed).

**TABLE 3:** Univariate analysis of age at onset and diagnostic delay with clinical manifestations.

	Clinical Manifestations													
	Motor developmental delay			Global developmental delay			Ataxia			Muscle tonic disorder				
	+	−	*p*	+	−	*p*	+	−	*p*	Normal	Hypotonia	Hypertonia	Dystonia	*p*
*Age at onset (month)*														
< 6	45	2	0.881	35	12	0.582	27	20	**0.003**	9	21	16	1	0.194
6–12	23	1		14	10		12	12		4	10	7	3	
13–36	17	0		11	6		4	13		7	4	6	0	
> 36	12	0		8	4		2	10		4	4	2	2	
*Diagnostic delay (months)*														
< 6	24	0	**0.023**	21	9	0.961	14	16	0.785	5	13	11	1	0.254
6–12	12	0		8	4		6	6		4	2	6	0	
13–36	33	0		23	10		14	19		10	14	8	1	
> 36	24	3		17	10		12	15		5	11	7	4	

*Note:* +, present; −, absent; Data are presented as number. Bold coefficients indicate statistical significance at *p* <0.05 (tow‐tailed).

No significant associations were observed between diagnostic delay and demographic factors (gender, prematurity, birth weight, family history), nor between baseline characteristics and neuroimaging/genetic findings. Furthermore, biological categories, atrophy topography, and clinical manifestations were not significantly correlated (Table 4).

**TABLE 4:** Univariate analysis of functional biological categories and neuroimaging features with clinical manifestations.

	Clinical Manifestations													
	Motor developmental delay			Global developmental delay			Ataxia			Muscle tonic disorder				
	+	−	*p*	+	−	*p*	+	−	*p*	Normal	Hypotonia	Hypertonia	Dystonia	*p*
*Functional categorization of biological abnormalities*														
EnzGly	14	0	0.915	7	7	**0.022**	5	9	0.305	3	5	6	0	0.439
DNA/RNAdef	26	1		22	5		12	15		5	12	8	2	
LyEnCy	8	0		5	3		5	3		3	1	4	0	
Channopathy	22	2		11	13		8	16		6	9	7	2	
MetabDis	9	0		9	0		7	2		1	5	3	0	
Transporter	6	0		5	1		2	4		0	5	1	0	
Cytoskeletal	13	1		10	4		7	7		6	3	3	2	
*Degree of atrophy*														
Mild	34	3	0.204	20	17	0.084	15	22	0.317	9	18	9	1	0.675
Moderate	35	0		26	9		14	21		9	12	12	2	
Severe	29	1		23	7		17	13		6	10	11	3	
*Topographic pattern (Abbr.)*														
iCA	34	0	0.085	21	13	0.565	14	20	0.189	8	15	9	2	0.871
CA + WM	21	1		15	7		10	12		4	8	8	2	
CA + CD	18	3		16	5		6	15		6	7	7	1	
CA + DG	14	0		8	6		9	5		4	3	6	1	
MSys	11	0		9	2		7	4		2	7	2	0	

*Note:* Data are presented as number; +, present; −, absent; Molecular classifications were abbreviated as follows: EnzGly, enzymopathy & glycoprotein disorders; DNA/RNAdef, DNA/RNA processing defects; LyEnCy, lysosomal, energy metabolism, and cytokine disorders; Channopathy, ion channel dysfunction; MetabDis, metabolic diseases; Transporter, membrane transporter defects; Cytoskeletal, cytoskeletal proteinopathies. Topographic patterns of cerebellar atrophy were classified as follows: CA, Cerebellar atrophy ± dentate nucleus/white matter involvement; CA + WM, CA with supratentorial white matter lesions; CA + CD, CA with cortical dysplasia; CA + DG, CA with basal ganglia, thalamic, or brainstem abnormalities (groups merged); MSys, multisystem degeneration (≥ 2 extracerebellar involvements). Bold coefficients indicate statistical significance at *p* <0.05 (tow‐tailed).

Multivariate regression analysis revealed that positive family history significantly predicted hypertonia over hypotonia (OR = 4.14, *p* = 0.042) or dystonia (OR = 19.33, *p* = 0.005). Similarly, enzymopathy/glycoprotein disorders independently predicted global developmental delay relative to cytoskeletal proteinopathies (OR = 4.4, *p* = 0.042). Early disease onset (≤ 6 months) was also a strong independent predictor of ataxia (OR = 6.75 vs. > 36 months, *p* = 0.021) (Table 5).

**TABLE 5:** Individual predictors of clinical outcomes from multiple regression models.

Clinical outcome	Predictor (comparison)	OR (95% CI)	*p*
Hypertonia	Positive family history		
	(ref: Hypotonia)	4.14 (1.05–16.35)	0.042
	(ref: Dystonia)	19.33 (2.52–148.20)	0.005
GDD	Enzymopathy & Glycoprotein (ref: Cytoskeletal)	4.40 (1.65–11.74)	0.042
Ataxia	Early onset (≤ 6 months) (ref: > 36 months)	6.75 (2.15–21.18)	0.021

*Note:* Each row represents a separate multivariate logistic regression model. Only variables with significant univariate associations are presented.

### 3.3 Neurodevelopmental Assessment

Neurodevelopment was assessed using age‐standardized instruments: the Peabody Developmental Motor Scales and Gesell Developmental Schedules for children under 6 years (Chałupka‐Borowska and Sobieska 2025), and the Wechsler Intelligence Scale for Children (WISC) for participants aged 6–16 years. Gross motor function was significantly associated with the type of biological dysfunction. Specifically, patients with membrane transporter defects (26.78 ± 17.90) or metabolic diseases (28.09 ± 26.26) exhibited lower gross motor scores than those with ion channel dysfunction (44.99 ± 14.97; *p* = 0.038) or cytoskeletal proteinopathies (47.81 ± 13.55; *p* = 0.027) (Table 6).

**TABLE 6:** Univariate analysis of developmental scores and associated factors.

	Developmental scores							
	Gross motor	*p*	Fine motor	*p*	Cognitive	*p*	Language	*p*
*Functional categorization of biological abnormalitie*								
EnzGly	34.32 ± 20.22	**0.033**	39.92 ± 18.77	0.103	44.42 ± 21.05	0.393	42.55 ± 17.21	0.148
DNA/RNAdef	43.81 ± 17.90		46.38 ± 18.68		47.58 ± 16.52		45.22 ± 17.63	
LyEnCy	34.38 ± 10.53		35.00 ± 10.69		38.31 ± 8.45		41.13 ± 9.66	
Channopathy	44.99 ± 14.97		49.48 ± 17.47		50.69 ± 17.65		52.75 ± 18.20	
MetabDis	28.09 ± 26.26		36.19 ± 24.17		44.97 ± 25.87		55.54 ± 26.98	
Transporter	26.78 ± 17.90		30.58 ± 13.65		32.79 ± 20.55		38.04 ± 21.76	
Cytoskeletal	47.81 ± 13.55		50.33 ± 17.81		50.78 ± 19.29		57.60 ± 21.57	
*Age at onset (month)*								
< 6	30.78 ± 15.65	**< 0.001**	35.61 ± 16.71	**< 0.001**	37.94 ± 16.12	**< 0.001**	42.03 ± 17.44	**0.010**
6–12	47.97 ± 16.41		49.76 ± 19.47		54.43 ± 18.90		54.05 ± 19.80	
13–36	44.12 ± 17.64		51.97 ± 16.36		50.91 ± 16.16		49.69 ± 19.52	
> 36	55.22 ± 17.21		53.78 ± 15.20		57.33 ± 19.11		62.11 ± 18.96	
*Diagnostic delay (months)*								
< 6	39.62 ± 17.83	**0.041**	43.69 ± 18.57	**0.034**	46.22 ± 18.74	**0.046**	48.70 ± 19.75	0.478
6–12	40.81 ± 18.12		44.67 ± 18.38		47.41 ± 18.37		49.13 ± 19.61	
13–36	40.26 ± 18.21		44.08 ± 18.46		46.55 ± 18.68		48.60 ± 19.73	
> 36	40.02 ± 17.54		44.27 ± 18.32		46.96 ± 18.43		48.44 ± 18.99	
*Degree of atrophy*								
Mild	47.36 ± 20.17	**0.018**	49.18 ± 18.09	0.089	51.99 ± 18.63	0.064	53.04 ± 20.23	0.078
Moderate	37.06 ± 13.85		41.78 ± 16.37		44.72 ± 17.05		49.08 ± 17.70	
Severe	35.27 ± 18.64		40.55 ± 20.82		42.32 ± 19.52		43.07 ± 19.63	

*Note:* Data are presented as mean ± SD. Bold coefficients indicate statistical significance at *p* <0.05 (tow‐tailed).

Patients with complex presentations scored lower in the gross motor (29.83 ± 18.29 vs. 44.44 ± 16.90, *p* = 0.029) and fine motor domains (32.94 ± 13.42 vs. 48.51 ± 17.91, *p* = 0.020) than those with isolated cerebellar atrophy. The complex group showed lower cognitive scores than patients with cerebellar atrophy combined with brainstem or basal ganglia/thalamic involvement (37.25 ± 11.19 vs. 53.34 ± 19.15, *p* = 0.019). Additionally, patients with cerebellar atrophy plus supratentorial cortical atrophy demonstrated poorer language function than those with isolated cerebellar atrophy (40.32 ± 15.66 vs. 51.50 ± 19.02, *p* = 0.042).

**FIGURE 2:**
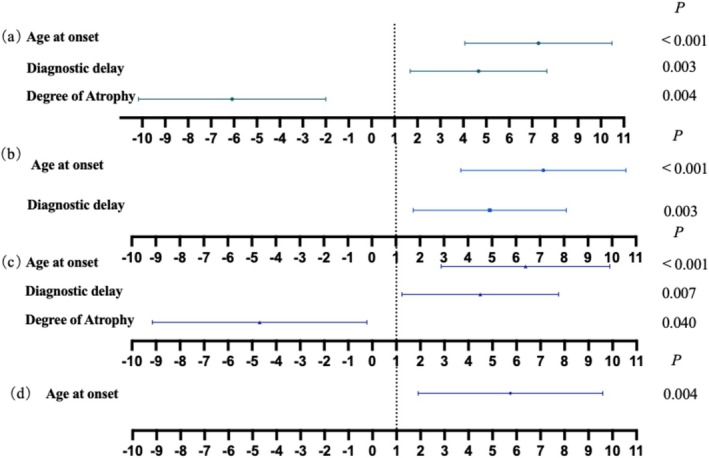
Forest plots of multivariable regression for the four functional domains. Forest plots representing unstandardized coefficients (B) and their 95% confidence intervals of predictor variables on (a) gross motor, (b) fine motor, (c) cognitive, and (d) language outcomes, calculated using multivariable regression analysis. *p*‐values were obtained from multivariable regression analysis.

Multivariable regression analysis identified several independent predictors of neurodevelopmental outcomes across functional domains. As illustrated in Figure 2, which presents the unstandardized coefficients (B) and 95% confidence intervals for all analyzed variables, three key factors demonstrated significant associations: Earlier age at onset consistently predicted poorer outcomes across all four functional domains, showing positive standardized coefficients (Beta) for gross motor (+0.412, *p* < 0.001), fine motor (+0.393, *p* < 0.001), cognitive (+0.349, *p* < 0.001), and language function (+0.300, *p* = 0.004). Longer diagnostic delay was associated with reduced scores in gross motor (+0.282, *p* = 0.003), fine motor (+0.291, *p* = 0.003), and cognitive domains (+0.265, *p* = 0.007). Greater cerebellar atrophy severity specifically predicted poorer gross motor (−0.270, *p* = 0.004) and cognitive outcomes (−0.203, *p* = 0.040). These findings indicate distinct pathological mechanisms: while earlier onset and longer diagnostic delays affect multiple developmental domains, cerebellar atrophy severity shows selective impact on motor and cognitive functions.

## 4 Discussion

Hereditary cerebellar atrophy (HCA) in children constitutes a group of rare neurological disorders characterized by complex phenotypes and high genetic heterogeneity. It is defined by progressive degeneration of the cerebellum and its associated pathways, with core clinical manifestations comprising cerebellar syndrome, often accompanied by additional neurological or non‐neurological impairments. Despite advancements in molecular diagnostics that have improved the efficiency and cost‐effectiveness of identifying genetic causes of cerebellar atrophy (CA), achieving timely diagnosis and effective treatment remains challenging. Large‐sample studies delineating the etiology and clinical spectrum of CA are scarce, with the most comprehensive reference dating to 2015 (Poretti et al. 2015). To address this gap, we conducted an integrated analysis of demographic, clinical, neuroimaging, neuropsychological, and genetic data from a Chinese pediatric HCA cohort, aiming to elucidate the complex interrelationships among these multidimensional factors.

Prematurity and negative family history significantly predicted earlier disease onset. Genetically mediated cerebellar atrophy may predispose to preterm birth, likely due to genetic defects disrupting embryonic development (Cossée et al. 2000). Preterm delivery itself can impair cerebellar maturation and neural connectivity, potentially explaining earlier clinical presentation (Xu et al. 2024). However, cerebellar atrophy in preterm infants is more commonly associated with acquired lesions (e.g., cerebellar hemorrhage) than with genetic causes. Although we excluded patients with documented perinatal brain injury, residual confounding and mild nonspecific imaging findings may still introduce bias, and thus these results should be interpreted with caution. In patients without a family history, early and severe manifestations may stem from de novo mutations causing severe protein dysfunction; the absence of familial clues often lowers clinical suspicion and delays diagnosis. Furthermore, motor developmental delay was universally observed in males and significantly more prevalent than in females. This disparity may reflect extended vulnerability of cerebellar development, delayed maturation, and prolonged formation of neural circuits in males (Sathyanesan et al. 2019; Tiemeier et al. 2010; Wang et al. 2023; Lyu et al. 2025), suggesting increased susceptibility to early motor impairment in male infants.

In summary, we recommend that a lower threshold for neuroimaging should be considered in high‐risk subgroups (preterm infants, males, and those with unremarkable perinatal or family histories), particularly when early non‐specific developmental red flags (e.g., motor delay or hypotonia) are present, rather than waiting for classic ataxia to emerge. This targeted approach may help reduce diagnostic delay, but should be guided by clinical judgment and does not imply universal screening. This recommendation is based on our MRI‐positive cohort and should be interpreted with caution, as we lack data on MRI‐negative children with similar clinical presentations. Earlier disease onset predicts more severe neurological impairment and poorer outcomes, underscoring the time‐sensitive nature of cerebellar development. Spatiotemporal maturation heterogeneity sees the vermis mature by age ~8 years, while anterior and superior posterior regions develop into adolescence (Tiemeier et al. 2010; Hodgdon et al. 2024). Concurrently, ongoing myelination renders early childhood a vulnerable period; disease onset during this window disrupts both structural and myelin maturation, culminating in significant motor and cognitive deficits (Durston et al. 2001; Kaczkurkin et al. 2019).

Cerebellar atrophy is commonly accompanied by ataxia (Martínez‐Rubio et al. 2023; Chemin et al. 2018). However, ataxia was not a predominant feature in our cohort except among patients with early onset (≤ 6 months). This may be attributed to the young age of most patients (hindering ataxia assessment), masking by coexisting movement disorders (e.g., spasticity, dystonia), and underreporting inherent to the retrospective design and absence of validated scales. Earlier onset was associated with more severe cerebellar dysfunction and more apparent classical ataxic symptoms. These findings highlight the importance of monitoring non‐ataxic manifestations (e.g., hypotonia and motor delay) in early‐onset cases to avoid diagnostic oversight. No regional predilection was observed in atrophy distribution; however, both the extent and complexity of atrophy correlated with neurological impairment. Specifically, patients with complex atrophy showed worse motor function than those with isolated cerebellar involvement, likely due to relative preservation of supratentorial motor regions in the latter group (Hogeveen et al. 2022; Li et al. 2022). Similarly, language deficits were more severe in cases with additional supratentorial cortical atrophy, suggesting functional disruption of Broca's and Wernicke's areas (Diedrichsen et al. 2019). We recommend individualized neuroimaging‐based assessment: prioritizing motor evaluation in isolated atrophy and incorporating language assessment when supratentorial involvement exists. Greater atrophy extent predicted worse motor and cognitive outcomes, indicating poorer prognosis (Yang et al. 2019; Steiner et al. 2023).

The type of biological dysfunction was a significant determinant of global developmental delay (GDD). Enzymopathies and glycoprotein disorders posed a higher risk of GDD compared to cytoskeletal proteinopathies, likely due to their broader disruption of essential metabolic pathways critical for neurodevelopment (Ligezka et al. 2021; Gallego et al. 2024; Mangione et al. 2025). Consequently, metabolic screening for these disorders should be prioritized, especially given the existence of targeted treatments for specific inborn errors of metabolism.

Gross motor function scores varied significantly across etiological subtypes, with cytoskeletal proteinopathies demonstrating the highest scores and membrane transporter defects or metabolic diseases the lowest. This discrepancy likely reflects the more complex pathophysiology and rapidly progressive clinical course characterizing the latter groups—for instance, SLC6A1 mutations disrupting synaptic inhibition and causing early‐onset ataxia (Kahen et al. 2022; Silva et al. 2024), or mitochondrial energy defects leading to neuronal failure and motor decline (Fernández de la Torre et al. 2023). Although these disorders generally confer a poor prognosis, certain subtypes are amenable to early intervention, such as miglustat or N‐acetyl‐L‐leucine for Niemann–Pick type C (Freihuber et al. 2023; Bremova‐Ertl et al. 2024), or ketogenic diets for pyruvate dehydrogenase complex deficiency (Li et al. 2024). Thus, early genetic and metabolic screening followed by targeted interventions (e.g., substrate‐specific diets or metabolite replacement) may improve motor outcomes in these children.

This retrospective case‐series study has several limitations, including the lack of standardized quantitative assessments for ataxia and neuroimaging characteristics, the difficulty of distinguishing true atrophy from mild nonspecific findings (e.g., mild vermian hypoplasia) on single MRI, particularly in younger children and preterm infants, and the absence of long‐term follow‐up data. Our cohort also includes non‐CA‐core genetic disorders, increasing heterogeneity. Moreover, we did not include an MRI‐negative control group with similar clinical presentations, and most hereditary CA lack disease‐modifying therapies. Therefore, the value of early neuroimaging lies mainly in shortening diagnostic delay and enabling genetic counseling rather than directly altering long‐term outcomes.

This study identified preterm birth, male sex, and negative family history as risk factors for earlier onset, emphasizing the need for early neuroimaging to avoid diagnostic delay. Early onset correlated with more severe motor, language, and cognitive deficits, indicating a poorer prognosis. Although ataxia was uncommon overall in this pediatric CA cohort, it occurred more frequently in patients with onset ≤ 6 months, necessitating attention to non‐ataxic presentations. Cerebellar atrophy showed no specific regional pattern, yet complex and severe atrophy were associated with significant motor impairment and poor prognosis. Although no predominant genetic or pathological hotspot was identified, metabolic disorders exhibited particularly severe motor dysfunction. Given the treatability of some metabolic subtypes, early metabolic and genetic screening is recommended to guide intervention.

## Author Contributions

Luyao Jin and Shuling Chen jointly conducted the data analysis and prepared the manuscript, and are listed as co‐first authors of this study. Ying Liu and Renyi She collected and analyzed the clinical and laboratory data of the pediatric patients. Wei Jiang served as the corresponding author of this article, who designed and supervised the study. All authors have read and approved the final manuscript.

## Funding

This work was supported by the National Natural Science Foundation of China (Grant/Award Number: 81571091), and the Chongqing Science and Technology Commission and Health Commission Joint Research Project (Grant/Award Number: 2018ZDXM029).

## Ethics Statement

This study has been approved by the Ethics Committee of the Children's Hospital affiliated with Chongqing Medical University (Approval No. 2023‐349) and informed consent has been obtained from the subjects and their families.

## Consent

The publication of all individual clinical information, imaging pictures, and facial images included in this article has been consented to by the parents of the pediatric patients. All authors have read and approved the final manuscript.

## Conflicts of Interest

The authors declare no conflicts of interest.

## Supporting information


**Figure S1:** Brain MRI images of 3 patients with CA. (a, b) Mild, (c, d) moderate, and (e, f) severe cerebellar atrophy.

## Data Availability

The datasets used and analyzed during the current study are available from the corresponding author on reasonable request.
