# Supplementary material for: Correlation Analysis of Clinical, Imaging, and Genetic Etiologies in Pediatric Hereditary Cerebellar Atrophy: A Single‐Center Study
Source: Mol Genet Genomic Med. 2026 Jul 6;14(7):e70258. doi: 10.1002/mgg3.70258 (PMC13338570; doi:10.1002/mgg3.70258)
Supplement: Supplementary file 1 — Figure S1: Brain MRI images of 3 patients with CA. (a, b) Mild, (c, d) moderate, and (e, f) severe cerebellar atrophy. [file MGG3-14-e70258-s001.docx]

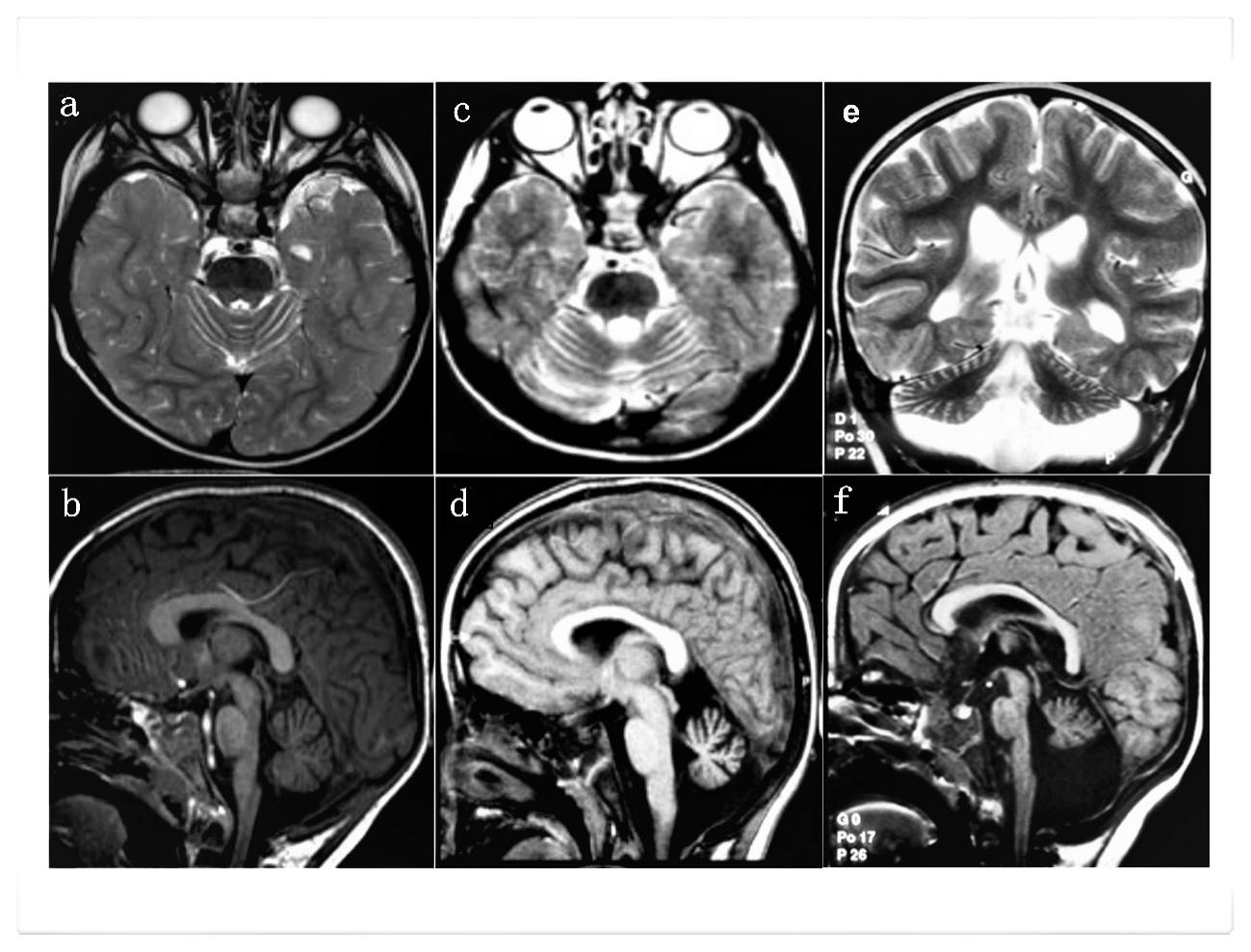


FigureS1 Brain MRI images of 3 patients with CA

(a,b) Mild, (c,d) moderate, and (e,f) severe cerebellar atrophy
